# Vulnerability to HIV infection among sex worker and non-sex worker female injecting drug users in Dhaka, Bangladesh: evidence from the baseline survey of a cohort study

**DOI:** 10.1186/1477-7517-3-33

**Published:** 2006-11-17

**Authors:** Tasnim Azim, Ezazul I Chowdhury, Masud Reza, Munir Ahmed, Mohammed T Uddin, Repon Khan, Giasuddin Ahmed, Motiur Rahman, Irona Khandakar, Sharful I Khan, David A Sack, Steffanie A Strathdee

**Affiliations:** 1 HIV/AIDS Programme, International Centre for Diarrhoeal Disease Research, Bangladesh (ICDDR,B), Mohakhali, Dhaka 1212, Bangladesh; 2 HIV Programme, CARE, Bangladesh, Dhaka, Bangladesh; 3 Division of International Health and Cross Cultural Medicine, Department of Family and Preventive Medicine, University of California San Diego School of Medicine, USA

## Abstract

**Background:**

Very little is known about female injecting drug users (IDU) in Bangladesh but anecdotal evidence suggests that they are hidden and very vulnerable to HIV through both their injection sharing and sexual risk behaviors. In order to better understand the risks and vulnerability to HIV of female IDU, a cohort study was initiated through which HIV prevalence and risk behaviors was determined.

**Methods:**

All female IDU (those who had injected in the last six months and were 15 years or older) who could be identified from three cities in the Dhaka region were enrolled at the baseline of a cohort study. The study was designed to determine risk behaviors through interviews using a semi-structured questionnaire and measure prevalence of HIV, hepatitis C and syphilis semiannually. At the baseline of the cohort study 130 female IDU were recruited and female IDU selling sex in the last year (sex workers) versus those not selling sex (non-sex workers) were compared using descriptive statistics and logistic regression.

**Results:**

Of the 130 female IDU enrolled 82 were sex workers and 48 were non-sex workers. None had HIV but more sex workers (60%) had lifetime syphilis than non-sex workers (37%). Fewer sex worker than non-sex worker IDU lived with families (54.9% and 81.3% respectively), but more reported lending needles/syringes (29.3% and 14.6% respectively) and sharing other injection paraphernalia (74.4% and 56.3% respectively) in the past six months. Although more sex workers used condoms during last sex than non-sex workers (74.4% and 43.3% respectively), more reported anal sex (15.9% and 2.1% respectively) and serial sex with multiple partners (70.7% and 0% respectively). Lifetime sexual violence and being jailed in the last year was more common in sex workers.

**Conclusion:**

Female IDU are vulnerable to HIV through their injection and sexual risk behaviors and sex worker IDU appear especially vulnerable. Services such as needle exchange programs should become more comprehensive to address the needs of female IDU.

## Background

Explosive HIV epidemics have occurred in many countries in injecting drug users (IDU), most recently in Asia [1], where risky injection practices have been attributed to rapid HIV spread. However, beyond needle/syringe sharing behaviors, sexual behaviors are strongly associated with HIV infection in both male and female IDU, particularly in countries where injection risks have been reduced [2]. This issue is further compounded by an overlap of sex and drug networks of IDU, which may not only enhance the vulnerability to HIV among IDU but can promote HIV transmission among IDU's sexual partners [3].

The experience of drug dependence is often different for women than for men [4] and risk factors for HIV infection among IDU differ significantly by gender [3,5,6]. Among female IDU, risky sexual behaviors (e.g., sex trade, having a male IDU sex partner, having an STI) can predominate as risk factors. Many female IDU sell sex in exchange of money or drugs [7,8] and studies have shown that female IDU involved in selling sex are more vulnerable to HIV [5,7]. Sexual violence has also been found to be associated with HIV infection in IDU and this is more commonly reported by female IDU [9]. These gender differences need to be better understood within a cultural context in order to inform effective HIV prevention programs.

In Bangladesh, HIV infection rates are still low [10] but is now on the rise in some populations. For example, in Central Bangladesh, 4.9% of male IDU tested HIV positive in 2005 [10]; in one neighborhood of the capital city, Dhaka, 8.0% of male IDU tested HIV positive in 2004 [11]. The reasons for these low rates are not clearly understood and it is believed that the needle/syringe exchange program (NSEP) which started early in Bangladesh may have played a role in this [12]. However, this is controversial as data from the National Behavior Surveillance Survey (BSS) showed that 77.2% of male IDU from Central Bangladesh reported borrowing and/or lending used syringes the last time they injected [13]. Risky sexual behaviors were also found to be highly prevalent with more than one third of male IDU from Central Bangladesh reporting non-commercial sex and 34.5% reporting buying sex in the last year [13] most without using condoms. Such risky behaviors persisted despite the presence of an active NSEP that also distributes condoms [13,14]. Very few female IDU have been reached in Bangladesh [15]. The evidence available is largely anecdotal suggesting that these women are even more marginalized than male IDU and that a high proportion may be involved in the sex trade.

In Dhaka, the capital city of Bangladesh, prevention programs have been operated by an international non-governmental organization (NGO), CARE, Bangladesh for female street based sex workers since 1997 and for IDU since 1998. Harm reduction services including NSEP and oral drug substitution are not legal in Bangladesh, [16] but despite this CARE, Bangladesh runs a NSEP which is operated primarily through outreach workers and has several drop-in centers within the community through which they provide services for sexually transmitted infections (STI), abscess management, rest and recreational facilities, HIV/AIDS education, needle/syringe exchange and male condoms. In addition, two week detoxification camps are organized at regular intervals. However, these well established services are mainly provided to male IDU [14]. In 2002, CARE Bangladesh began offering NSEP to a small number of female IDU; in 2004, two drop-in centers were opened specifically for female IDU and 28 women have undergone detoxification. HIV prevention services are provided to female sex workers in the streets [17] through a network of outreach services providing services similar to that described above with the exception of drug related services. In addition, there is a self-help group of female sex workers, which actively promotes HIV prevention and human rights.

Given the present situation in Bangladesh – high levels of risk behaviors, lack of a legal framework for the operation of harm reduction services and the early stages of the HIV epidemic, an explosive HIV epidemic appears to be imminent.

Female IDU are often doubly vulnerable to the HIV epidemic and in order to better understand the vulnerability of female IDU to HIV in Bangladesh, a cohort study was initiated in three cities in the Dhaka region. As a large proportion of those female IDU reached were sex workers, and as research in some countries has shown that the risks and vulnerabilities of the sex worker and non-sex worker IDU can be different [8,18] the present study aimed to assess whether risk behaviors differed for female IDU who reported sex work, relative to those that did not. Such findings have important implications for program planning in Bangladesh and elsewhere in South Asia, where the number of female IDU appears to be increasing.

## Methods

### Study participants

Between December 2004 and May 2005, female IDU were enrolled into a cohort study from three cities in the Dhaka Division – Dhaka, the capital city, Tongi (27 km to the north of Dhaka city) and Narayanganj (23 km to the south east of Dhaka city). All women 15 years and older with a history of injecting drugs at least once in the last six months were eligible for enrolment. Enrolment of female IDU was non-random and included those who were accessed with the help of outreach workers from the NSEP of CARE, Bangladesh, and by snowballing through the networks of female and male IDU, female heroin smokers and that of female sex workers.

### Study measures

After obtaining informed consent, trained staff administered a semi-structured questionnaire to the female IDU at the field sites. The risk behavior questionnaire was designed to ascertain demographic characteristics, drug use characteristics, injection and sexual risk behaviors (over their lifetime, in the last six months, the last month and last week), experiences of sexual abuse, incarceration, knowledge about HIV/AIDS and care seeking practices. Interviews were conducted by one male and one female interviewer at field sites where some privacy could be maintained.

Women were then requested to attend the drop-in center of CARE, Bangladesh where a female clinician conducted physical examinations and blood was drawn for HIV, syphilis and hepatitis C (HCV) antibody testing. Female IDU who did not wish to attend the drop-in center were referred to the Voluntary Counseling and Testing (VCT) Unit of the International Centre for Diarrhoeal Disease Research, Bangladesh (ICDDR, B).

Following the interview, a 5 ml blood sample was collected by venepuncture and transported to the Virology Laboratory of ICDDR, B, while maintaining the cold chain. Serum was separated and stored at -20°C until testing was done. Syphilis was tested by the Rapid Plasma Reagin (RPR) test (Nostion II, Biomerieux BV, Boxtel, The Netherlands) and Treponema Pallidum Particle Agglutination (TPPA) test (Serodia TPPA, Fujirebio Inc., Japan). Samples positive by TPPA with an RPR titer of  <1:8  were considered as positive while those positive for TPPA with an RPR  titer of ≥1:8 were considered to reflect active syphilis. Antibodies to HCV were initially tested using an Enzyme Linked Immunosorbent Assay (ELISA) kit (UBI HCV EIA 4.0, United Biomedrop-in centeral Inc, USA and Hepanostika HCV Ultra, Beijing United Biochemical Co. Inc., Ltd., Beijing, PR China) and positive samples were re-tested with a second ELISA kit (IM_X _HCV Version 3.0, Abbott Laboratories, IL, USA). Discrepant results were confirmed by Line Immunoassay (LIA; INNO-LIA HCV, Innogenetics, Ghent, Belgium). Samples positive for any two tests were considered HCV antibody positive. For HIV antibody testing, samples were initially tested by a commercial ELISA kit (Vironostika HIV Uni-Form II Plus O, Biomerieux, Boxtel, the Netherlands) and positive results were confirmed by LIA (INNO-LIA HIV I/II Score, Innogenetics).

All test results were provided to the female IDU with post-test counseling. Treatment for syphilis was provided according to the National STI Management Guidelines in Bangladesh. For other diseases, treatment was provided through available services of CARE, Bangladesh. The study protocol was approved by the Ethical Review Committee of ICDDR, B.

### Data analysis

Female IDU enrolled were then divided into two groups of women; those who had sold sex in exchange of money or drugs within the last year (sex worker IDU) and those who had not sold sex within the last year (non-sex worker IDU). Descriptive analyses were conducted by running frequency tables, calculating means and medians. For categorical variables, exact binomial confidence intervals were calculated. Comparisons between IDU reporting and not reporting commercial sex work were conducted using the Chi-square test and the Mann-Whitney U test. Univariate logistic regression was performed to assess the extent to which female IDU reporting sex work were engaging in riskier behaviors than other female IDU.

## Results

### Demographic characteristics

A total of 135 female IDU were enrolled in the cohort study; however, sexual histories were incomplete on five women, who were hence excluded from these analyses. Of the remaining 130 female IDU, 82 (63.1%) had sold sex in the last year (sex worker female IDU) and 48 (36.9%) had not (non-sex worker female IDU). Most women were from Dhaka (78.5%), 17.7% were from Tongi and only five (3.8%) were from Narayanganj.

The median ages of the female IDU sex workers (median = 27.0 years, interquartile range [IQR]: 23.0–33.3 years) and non-sex workers (median = 30.0 years, IQR: 24.0–39.5 years) were similar. As shown in Table 1, similar proportions of sex workers and non-sex workers were married (42.7% and 62.5% respectively), however, sex workers (54.9%) were less likely to be living with their relatives (including spouse) compared to non-sex workers (81.3%) (OR: 0.3, 95% CI: 0.1–0.7). Although more sex workers (34.1%) lived on the streets compared to non-sex workers (22.9%), the difference was not statistically significant.

**Table 1:** Demographic characteristics and needle/syringe and drug sharing behavior among female IDU reporting and not reporting sex work in Bangladesh

**Characteristics**	**Sex workers (%) (N = 82 unless otherwise stated)**	**Non-sex workers (%) (N = 48 unless otherwise stated)**	**Odds Ratio**	**(95% CI)**
Marital status				
Currently married	42.7	62.5	0.5	0.2–1.0*
Unmarried	4.9	2.1	2.4	0.2–58.4
Separated/divorced/widow	52.4	35.4	2.0	0.9–4.5
Mostly living with				
Relatives (including spouse)	54.9	81.3	0.3	0.1–0.7**
Friends	6.1	2.1	3.1	0.3–71.2
Other drug users	11.0	4.2	2.8	0.5–20.0
Alone	28.0	12.5	2.7	0.9–8.2*
Current living area				
Slum^†^	50.0	50.0	1.0	0.5–2.2
Residential area (not slum)^§^	13.4	25.0	0.5	0.2–1.3
Work place	1.2	2.1	0.6	0–21.8
On the street	34.1	22.9	1.7	0.7–4.3
Mazar^‡^	1.2	0		
Ever shared needles/syringes	96.3	85.4	4.5	1.1–18.3*
Borrowed used needles/syringes during last injection in the last 6 months	30.5	22.9	1.5	0.6–3.4
Lent used needles/syringes during last injection in the last 6 months	29.3	14.6	2.4	1.0–6.2*
Borrowed used needles/syringes in the last 6 months	72.0	62.5	1.5	0.7–3.3
Lent used needles/syringes in the last 6 months	70.7	66.7	1.2	0.6–2.6
Borrowed used needles/syringes in the last month	54.9	41.7	1.7	0.8–3.5
Lent used needles/syringes in the last month	52.4	41.4	1.5	0.8–3.2
Borrowed used needles/syringes in the last week	37.8	35.4	1.1	0.5–2.3
Lent used needles/syringes in the last week	39.0	33.3	1.3	0.6–2.7
Frequency of borrowing used needles/syringes in the last six months (among those who borrowed in the last 6 months)	N = 59	N = 30		
Always	20.3	13.3	1.6	0.5–5.7
Sometimes	79.7	86.7	0.6	0.2–2.3
Frequency of lending used needles/syringes in the last six months (among those who lent in the last 6 months)	N = 58	N = 32		
Always	17.2	6.3	3.1	0.6–15.3
Sometimes	82.8	93.8	0.3	0–1.7
Relationship with needles/syringes sharing partners (among those who had ever shared)	N = 79	N = 41		
Friend	29.1	31.7	0.9	0.4–2.0
Family member	2.5	9.8	0.2	0.04–1.4
Husband/lover	16.5	29.3	0.5	0.2–1.2
Acquaintances/strangers	55.7	39.0	2.0	0.9–4.2
Sources of obtaining needles/syringes				
NSEP	69.5	79.0	0.8	0.3–1.7
Pharmacy	50.0	47.9	1.1	0.5–2.2
Friends/other drug users	13.4	4.2	3.6	0.8–16.8
Shared injection paraphernalia while injecting drugs in the last 6 months	74.4	56.3	2.3	1.1–4.8*

* p value < 0.05, ** p value < 0.01

^†^Slum: characteristics include poor housing, high housing density, shared latrine, poor sewerage and drainage facility

^§^Residential area: better quality housing with independent kitchen and latrine facilities

^‡^Mazar: religious shrine

The percentage who reported no schooling or less than five years of schooling was similar for sex workers and non-sex workers (87.8% versus 89.6%, respectively). The median average monthly income reported for the last six months was higher for sex workers (US$ 71; IQR US$50–100) than for non-sex workers (US$ 43; IQR US$26–71) (p < 0.001). The earning for sex worker IDU is comparable to the BSS data from street recruited sex workers in Central Bangladesh [19]. The most common principal sources of income in the last six months were different for the two groups of women; amongst sex workers this was through selling sex (74.4%) and amongst the non-sex workers this was through selling drugs (27.1%). Sex worker IDU were less likely to sell drugs than non-sex workers (sex workers, 1.2% and non-sex workers, 27.1%; OR: 0.1, 95% CI: 0–0.6). Also, sex worker IDU were less likely to be receiving money from parents and other relatives compared to non-sex workers (sex workers: 2.4% and non-sex workers: 18.8%; OR 0.03; 95% CI: 0.0–0.3).

### Drug use characteristics

In both groups of female IDU, drugs used for injection were most commonly 'cocktails' of different pharmaceuticals, primarily buprenorphine with anti-histamines and sometimes diazepam. Although most women smoked heroin, injection of heroin was not common.

Most of the female IDU, whether sex workers or non-sex workers, reported that a member of their household used drugs (56.1% and 68.8%, respectively) and for most it was their husbands or other regular sex partner (sex workers: 78.3% and non-sex workers: 81.8%). Sex workers (30.5%) were more likely to report that they were inducted into drugs under the influence of friends compared to non-sex workers (6.3%) (OR: 6.6; 95% CI: 1.7–29.3). For non-sex workers the most common reason stated for starting drugs was curiosity (35.4%).

Before starting to inject, all but three women took drugs through routes other than injection, which included smoking cannabis, ingesting sedatives, inhaling heroin, ingesting codeine containing cough syrups, etc. The median duration for taking any kind of drugs was similar for the two groups of female IDU (sex workers: 7 years, IQR: 4.0–12.0 years and non-sex workers: 8 years, IQR: 3.4–14.3 years) and that for injecting drugs was two years for both groups. The most common cause cited for switching to injecting by both groups of female IDU was lower cost and easier availability of injection (43.9% and 58.3% respectively).

### Needle/syringe and drug sharing practices

Needle/syringe sharing (lending or borrowing) was common (Table 1). Compared to non-sex workers, sex worker IDU more commonly reported having ever shared their needles/syringes with others (OR: 4.5, 95% CI: 1.1–18.3) and lending their used needles/syringes during the last injection taken within the last six months (OR: 2.4, 95% CI: 1.0–6.2). However, irrespective of whether the IDU were sex workers or not, most of the women who shared needles/syringes reported doing so 'some of the time' in the last six months and they most commonly shared with acquaintances. Similar proportions of sex worker and non-sex worker IDU said that all their needle/syringe sharing partners were members of the NSEP (49.4% and 56.1% respectively). The average number of needle/syringe sharing partners during the last injection in the last year was two (IQR 2.0–2.0) for both groups of female IDU. In the last seven days, among those who shared needles/syringes, 73.5% of sex workers and 78.9% of non-sex workers reported sharing with the same people.

Other than needles/syringes, sex worker IDU were 2.3 times more likely to share injection paraphernalia than non-sex worker IDU (Table 1). At their last injection, almost all shared the ampoule that buprenorphine and other liquid pharmaceuticals are typically sold in.

Sex worker and non-sex worker IDU had other similarities in that similar proportions cleaned their syringes before sharing (data not shown) and they commonly used hot or cold water, some used paper/cloth/leaves to wipe the needles, which they considered as cleaning. Approximately half had devised their own ways of cleaning while close to one third had learnt these doubtful methods from friends.

The sources of needles/syringes for sex worker and non-sex worker IDU were similar, the most common being the NSEP. Of the respondents who were members of the NSEP and obtained needles/syringes from sources other than the NSEP, the main reasons cited were lack of NSEP availability.

### Sexual experiences and risk behavior

Comparison of sexual risk behavior and vulnerability between sex worker and non-sex worker female IDU is shown in Table 2. The median age at first sexual encounter was 14 years for both groups of female IDU (IQR: sex workers, 12.0–15.3 and non-sex workers, 12.0–16.0) and this was more likely to be the husband for non-sex workers compared to sex workers (77.1% and 40.2 %, respectively). However, in the case of sex workers, this encounter was three times more likely to be forced (Table 2).

**Table 2:** Sexual risk behavior and prevalence of HIV, syphilis and hepatitis C antibodies among female IDU reporting and not reporting sex work in Bangladesh

**Characteristics**	**Sex workers, (%) (N = 82 unless otherwise stated)**	**Non-sex workers, (%) (N = 48 unless otherwise stated)**	**Odds Ratio**	**(95% CI)**
Type of first sex act				
Consensual	56.1	79.2	0.3	0.1–0.8**
Forced	43.9	20.8	3.0	1.2–7.4**
Relationship with first sexual partner				
Husband	40.2	77.1	0.2	0.1–0.5**
Friend/lover	18.3	12.5	1.6	0.5–5.0
Clients	19.5	8.3	2.7	0.8–10.2
Mastans (hoodlums)	11.0	2.1	5.8	0.7–126.1
Types of sex acts practiced in their lifetime				
Vaginal	100	100		
Anal	15.9	2.1	8.9	1.1–70.0*
Oral	26.8	14.6	2.1	0.8–5.5
Had sex last year	100	62.5		
Used condom during last sex in the last year (among those who had anal/vaginal sex in the last year)	74.4	N = 30 43.3	3.8	1.6–9.1**
Currently having non-commercial sex partner	52.4	64.6	0.6	0.3–1.3
Relationship with current non-commercial sex partner(s) (among those who have current non-commercial sex partners)	N = 43	N = 31		
Husband	76.7	93.5	0.2	0.1–1.1
Boyfriend/lover	27.9	9.7	3.6	0.9–14.1
Had non-commercial sex partner in the last week	35.4	25.0	1.6	0.7–3.6
Frequency of using condoms with non-commercial sex partner in the last week (among those with non-commercial partners in the last week)	N = 29	N = 12		
Always	34.5	50.0	0.5	0.1–2.5
Sometimes	3.4	16.7	0.2	0.01–3.0
Never	62.1	33.2	3.3	0.7–17.2
Prevalence of HIV	N = 75 0	N = 46 0		
Prevalence of syphilis in lifetime (TPPA positive, RPR titer <1:8)	N = 75 60.0	N = 46 37.0	2.6	1.2–5.5*
Prevalence of active syphilis (TPPA positive RPR titer ≥1:8)	N = 75 10.7	N = 46 6.5	1.7	0.4–6.8
Prevalence of hepatitis C antibodies	N = 75 18.7	N = 46 13.0	1.5	0.5–4.3

* p value < 0.05, ** p value < 0.01

Among women who reported having sex in the last year, condom use during last sex was reported more commonly by sex workers (74.4%) than non-sex workers (43.3%; OR: 3.8, 95% CI: 1.6–9.1). Sex workers (15.9%) were 8.9 times more likely to have ever had anal sex than non-sex workers (2.1%).

Similar proportions of sex worker and non-sex worker IDU had current non-commercial sex partners at the time of interview (52.4% and 64.6%, respectively). Although a higher percentage of sex workers reported that they had never used condom during sex with those partners in the last week compared to non-sex workers (62.1% and 33.2%, respectively), the difference was not statistically significant.

Women were asked if they had had sex in the last year with several men at the same time (usually serially, one after another) and if yes, how many sex partners were involved in the last episode of such a "group sex" and whether she knew if any or none of the partners had used condoms during the last group sex episode. Group sex in the last year was reported only by the sex worker IDU (70.7% vs 0%, p < 0.001). The median number of partners in the last episode of group sex in the last month was 4.0 (IQR: 3.0–31.3) and during this episode, 20.7% of the female IDU reported that none of the partners used condoms.

When asked if they had shared needles/syringes with their sex partners, similar proportions of sex workers (28%) and non-sex workers (33.3%) reported doing so and similar proportions (34.8% and 37.5%) did not use condoms at their last sexual encounter with this person.

### Violence and experience of jail

Women were asked if they had experienced some form of violence (rape or other physical violence) ever in their lifetime. Sex workers were more likely to report experience of violence than non-sex worker IDU (78% and 27.1% respectively; OR: 9.6; 95% CI: 4.2–21.8). However, of those who had experienced violence, there were no differences in the proportions of women who were raped in the two groups (sex workers: 81.3% and non-sex workers: 61.5%). For sex workers the perpetrators of rape were most commonly strangers (40.4%) and mastans (hoodlums) (38.5%) while 50% (n = 4) of non-sex workers reported that it was their husbands who had committed the rape.

More sex worker (23.2%) than non-sex worker female IDU (8.3%) reported being in jail in the last year (OR; 3.3; 95% CI: 1.1–10.4). For sex workers the most common reason for being jailed was related to sex work (51%) while most non-sex workers (43.8%) were jailed under a special clause which authorizes the police to arrest anyone on 'reasonable suspicion' that s/he has or is about to commit a crime [20].

### Knowledge about HIV/AIDS

Female IDU were asked whether they had heard about HIV/AIDS, about routes of transmission, methods of protection and the source of their information. Sex worker and non-sex worker IDU were similar in that almost all had heard about HIV/AIDS (98.8% and 97.8%, respectively), most knew that sharing of needles/syringes (74.1% and 89.4%, respectively) and unprotected sex (77.8% and 78.7%, respectively) were important routes of HIV transmission. However, when asked about protection from transmission, sex workers were more likely to know that using condoms can protect against transmission compared to non-sex workers (86.4% and 68.1%, respectively; OR: 3.0, 95% CI: 1.2–7.2). In contrast, sex workers were less likely than non-sex workers to know that avoiding sharing of needles/syringes could be protective (65.4% and 85.1%, respectively; OR: 0.3; 95% CI: 0.1–0.8). On the whole, sex worker IDU (4.9%) were less likely to report incorrect means of protection from HIV than non-sex worker IDU (17.0%; OR = 0.3; 95% CI: 0.1–0.9).

The majority of the female IDU stated that their primary source of information on HIV/AIDS was NGOs and other service providers (sex workers: 90.1% and non-sex workers: 83.0%). Radio/television was less likely to be sources of information on HIV/AIDS for sex workers (19.8%) compared to non-sex workers (40.4%) (OR: 0.4; 95% CI: 0.2–0.8).

### Prevalence of blood-borne and sexually transmitted infections

Of the 130 women, 121 (93.1%) underwent clinical examinations and consented to giving blood. Four could not be traced, one had migrated out of the area and one was in hiding from law enforcement. In the case of one woman, a functional vein for drawing blood could not be found. Two refused to give blood although they completed their clinical examination.

None of the female IDU tested HIV positive (Table 2). Compared to non-sex worker IDU, sex worker IDU were more likely to have had syphilis antibodies reflecting infection at some point in their lives (RPR titer <1:8) although the percentage with active syphilis (RPR titer ≥1:8) were similar. HCV prevalence was also similar between sex worker and non-sex worker IDU.

### Services from the NSEP

Of the 130 female IDU, 22 (16.9%) did not use any of the services provided by CARE, Bangladesh that are available for either the IDU (i.e. NSEP) or sex workers. Of these 22, 13 knew of the services but did not use them and the reasons cited included: not wanting to disclose oneself as an IDU (n = 6), did not want to join (n = 4), was not asked to join (n = 3). Amongst the women who provided blood samples and underwent clinical examination, 26 (21.5%) refused to do so in the drop-in center of CARE, Bangladesh.

Although similar proportions of sex workers and non-sex workers (93.9% and 91.7%, respectively) knew about the services from CARE, Bangladesh, among those who knew, sex workers were more likely to have used those services than non-sex workers (93.5% and 81.8% respectively, OR: 3.2; 95% CI: 1.0–10.5). Among the non-sex worker IDU using services, all were exchanging needles/syringes while 79.2% of the sex workers were doing so (p < 0.01). On the other hand, sex workers were more likely to be receiving condoms than non-sex workers (79.2% vs. 38.9%; OR: 6.0; 95% CI: 2.5–14.4).

## Discussion

The links between sex work and injecting drug use have been shown to be important determinants in the spread of an HIV epidemic [21-23]. In Bangladesh, considerable risk behavior among male IDU has been documented through the annual Behavioral Surveillance Survey (BSS) [19] in whom the risks for HIV are not only through their risky injection practices but also their sexual behaviors. There is very limited information available about female IDU in the South and South East Asian region; most information is obtained through data on injection drug use in sex worker communities [24,25]. Similarly, in Bangladesh female IDU have been difficult to access and information is largely confined to drug taking behaviors among female sex workers from brothels, streets and hotels obtained from the BSS [19]. The female IDU enrolled in this study were identified through CARE, Bangladesh and through the networks of drug users and sex workers because of which sampling was not random and hence the data are not necessarily representative. Despite this limitation, this study is the first comprehensive report on female IDU in Bangladesh, which provides a comparative analysis of the risks and vulnerabilities of female IDU who do and do not sell sex.

This study revealed high levels of risk behavior and important similarities and differences in injecting and sexual risk behaviors for sex worker and non-sex worker female IDU. The study findings suggest that it is not only their individual behaviors but the circumstances that female IDU live in that can further marginalize and make them more vulnerable. We observed a substantial proportion of sex workers and non-sex workers were living on the streets which is pertinent to HIV as homelessness has been shown to be associated with higher HIV infection rates in IDU [26,27]. However, non-sex worker IDU were better off than sex worker IDU in this regard as they were more likely to be living with their relatives from whom they were receiving financial support. On the other hand, a higher proportion of non-sex workers were supporting themselves financially by selling drugs, which carries many risks including incarceration and exposure to violence.

The pattern of drug use between sex worker and non-sex worker female IDU described here was similar and this has also been reported from other countries [18]. However, riskier injection practices were documented among sex worker female IDU. Higher percentages of sex workers had shared needles/syringes ever in their lifetime, and more had shared their drug ampoule than non-sex workers. Such higher injection risks among sex worker female drug users have been reported from a study conducted among crack users in Kentucky, USA [7] but not in another conducted in IDU from Sydney, Australia [18]. Although we report that equal proportions of sex worker and non-sex worker IDU borrowed used needles/syringes in the recent past, sex workers were more likely to lend their used needles/syringes, indicating that sex worker IDU are not only more vulnerable themselves but their injection sharing partners are also at higher risk for blood borne infections.

It is indeed fortunate that in both groups of female IDU no HIV was detected. Although one may argue that we under-sampled higher risk female IDU, but the national HIV surveillance shows that Bangladesh is a low prevalence nation for HIV and that in 2005, there was no HIV detected in IDU (N = 2294) from 13 cities out of the 16 cities from where a total of 3682 IDU were sampled [10]. The reasons for this low prevalence are not clear although the NSEP may have played a role [12] especially as it commenced before any HIV was detected among IDU. However with rising HIV rates among IDU in Central Bangladesh there is no room for complacency [10] and it is essential that the harm reduction services are expanded, intensified with broad and active support from all relevant sectors.

We observed high risk sexual behaviors for both sex worker and non-sex worker groups of female IDU, and not surprisingly behaviors tended to be riskier for sex worker IDU. Although more sex worker IDU reported condom use during the last sex act, more reported anal sex, they had concurrent commercial and non-commercial sex partners and a substantial proportion had never used condoms with their non-commercial sex partners. Moreover, sex worker female IDU commonly reported serial sex with multiple partners (group sex), which was not reported at all by non-sex worker IDU. Although we were lacking data on the context of group sex and cannot assume that they were consensual, anecdotal reports suggest that group sex may occur within the context of sex work, with male clients pooling money to share a female sex worker. Such high levels of sexual risk behavior in female IDU are not unique to Bangladesh [28] and this has also been observed in places where IDU are accessing HIV prevention programs [29].

Consistent with the high risk sexual behaviors we observed, the prevalence of syphilis was high, especially among sex worker IDU who had a higher lifetime prevalence of syphilis. However, there was no significant difference in the prevalence of active syphilis between the two groups of female IDU which was also comparable to that reported by the national HIV surveillance data from street-recruited female sex workers in Central Bangladesh [13]. Although this study did not measure other STIs, other studies of sex workers from different sites in Bangladesh have recorded very high rates of the different STIs including gonorrhea, chlamydia, trichonomiasis, syphilis and herpes simplex 2 [30,31]. The sexual risk behaviors we documented among sex worker IDU are similar to those observed among female sex workers from Central Bangladesh reported in the BSS of Bangladesh [13]. However, compared to the BSS, the frequency of reported condom use we observed was higher. The reason for this discrepancy is not clear but we cannot rule out the possibility of socially desirable responding since this study was conducted in collaboration with CARE, Bangladesh.

Female IDU are often more vulnerable to HIV than their male counterparts due to greater overlap between sex and drug use networks [32]. Women who share drugs with their sex partners often share needles/syringes with these partners and may also have unprotected sex with them, compounding their risk of acquiring both blood-borne and sexually transmitted infections [3]. Close to one third of the female IDU in either group studied here reported having unprotected sex with their injection partners. This is of particular concern as male IDU in Central Bangladesh are at the brink of a concentrated HIV epidemic [10]. These women are not only extremely vulnerable to HIV but they may also represent 'transmission bridges' to the general community through commercial sex.

Sexual violence in IDU has been shown to be associated with greater risk of HIV infection and female IDU are more likely to have a history of sexual violence than males [9,33]. A higher percentage of sex worker IDU reported forced sex as their first sexual experience, compared to non-sex workers. Other studies have shown close associations between childhood sexual abuse, prostitution and early initiation into injection drug use [34].

In this study, sex worker IDU were more commonly jailed in the last year than non-sex worker IDU as has been shown in other studies [35]. For sex workers, the reason for incarceration was more frequently for selling sex while for non-sex workers it was associated with a special legal clause "section 54" which authorizes the police to arrest anyone on 'reasonable suspicion' that s/he has or is about to commit a crime without a warrant of arrest, or with the requirement to demonstrate any reasonable grounds for such suspicion [20]. Incarceration may pose an additional risk of HIV acquisition in jails where drugs are widely available but needles/syringes are not [27,36].

In this study, sampling of female IDU was non-random and most were associated with CARE, Bangladesh's intervention programs. This may explain why the overall knowledge about HIV transmission was high amongst both groups of female IDU. However, it was interesting that for prevention of transmission sex worker female IDU were more likely to mention condom use during sex while non-sex worker IDU were more likely to mention not sharing injection equipment as means of prevention. This knowledge pattern reflects the nature of services accessed by the two groups of female IDU, with sex workers more commonly availing of services for sex workers (i.e. condoms and STI management) whereas non-sex workers were more likely to access the NSEP. The services available for female IDU in Bangladesh are limited. The data here show that of the female IDU using the NSEP services several were getting their needles/syringes from other additional sources and the reasons provided for this indicated restrictions in access to NSEP. It has been well documented that effective harm reduction services including NSEP can reduce the spread of HIV in IDU [37,38] and with restricted access to the NSEP continued needle sharing has been recorded [39]. Modeling data obtained to assess the effectiveness of CARE Bangladesh's NSEP in Dhaka city suggests that the epidemic may have been blunted by the NSEP [12]. However, this view is controversial, as BSS data on IDU from Central Bangladesh do not show safer injection behaviors over time despite the presence of the NSEP [13].

## Conclusion

Female IDU in Bangladesh are at risk of a major HIV epidemic from both injection sharing and sexual risk behavior and sex worker IDU appear especially vulnerable. Once HIV enters this community the female IDU are likely to bridge the epidemic to the general population. The harm reduction services available to the female IDU are limited and our findings support the need to expand NSEP coverage to female IDU and to provide gender-sensitive harm reduction services, especially for those engaging in sex work. Similar to many countries in the region, although harm reduction services are not legal, NSEP is still active. This lack of legality makes it dangerous for the service providers as well as the beneficiaries hampering access to clean needles/syringes, condoms and other services. Oral drug substitution, which is non-existent in Bangladesh, could be a very effective harm reduction strategy for preventing the spread of HIV at this early stage of the epidemic.

## Authors' contributions

TA supervised all aspects of the study including data analysis, and drafted the manuscript

EIC supervised all field activities of the study and provided input to the manuscript

MR conducted the data analysis and provided input to the manuscript

MA supervised access to CARE facilities

MTU supervised access to IDU from CARE, Bangladesh

RK conducted HIV and hepatitis tests and analyzed the laboratory data

GA conducted syphilis tests and managed the laboratory data

MR supervised the syphilis tests

IK provided clinical services to the IDU and input to the manuscript

SIK provided input in the field supervision and preparation of the manuscript

DAS provided input to the manuscript

SAS provided input in the study design, analysis and drafting of the manuscript
